# Valorization of Olive Leaf Polyphenols by Green Extraction and Selective Adsorption on Biochar Derived from Grapevine Pruning Residues

**DOI:** 10.3390/antiox13010001

**Published:** 2023-12-19

**Authors:** Melissa Prelac, Nikola Major, Danko Cvitan, Dominik Anđelini, Maja Repajić, Josip Ćurko, Tvrtko Karlo Kovačević, Smiljana Goreta Ban, Zoran Užila, Dean Ban, Igor Palčić

**Affiliations:** 1 Institute of Agriculture and Tourism, Karla Huguesa 8, 52440 Poreč, Croatia; melissa@iptpo.hr (M.P.); danko@iptpo.hr (D.C.); dominik@iptpo.hr (D.A.); tvrtko@iptpo.hr (T.K.K.); smilja@iptpo.hr (S.G.B.); zoran@iptpo.hr (Z.U.); dean@iptpo.hr (D.B.); palcic@iptpo.hr (I.P.); 2 Department of Food Engineering, University of Zagreb, Faculty of Food Technology and Biotechnology, Pierottijeva 6, 10000 Zagreb, Croatia; maja.repajic@pbf.unizg.hr (M.R.); josip.curko@pbf.unizg.hr (J.Ć.)

**Keywords:** adsorption, bioactive compounds, green extraction, sustainable agriculture, waste management

## Abstract

Given today’s increasingly intensive agriculture, one key problem area considers the valorization and reuse of wastes from food and agricultural production with minimal impact on the environment. Due to its physicochemical characteristics, biochar (BC) derived from grapevine pruning residue has shown considerable potential for use as an adsorbent. High-value phytochemicals found in abundance in the olive leaf (OL) can be employed in many different industrial sectors. The potential application of BC in the removal of specific polyphenolic components from OL extracts has been investigated in the present study. Water, as the most available and greenest of solvents, was investigated as to its use in the extraction of polyphenols, which was carried out by comparing maceration, ultrasound-assisted extraction, and microwave-assisted extraction, considering different temperatures and solid-to-liquid (s/l) ratios. The BC adsorption capacity of selected polyphenols was fitted with both the Langmuir and Freundlich isotherm models. The Freundlich model fitted better relative to OL polyphenols adsorption. Oleuropein was the most abundant compound identified in the extracts, obtaining the highest K_f_ value (20.4 (mg/g) × (L/g)^n^) and R^2^ coefficient (0.9715) in the adsorption on the biochar’s surface. The optimum conditions in the dosage experiment suggest the use of 0.5 g of BC using 3 g/L extracts, with an exception for oleuropein and hydroxytyrosol, for which the highest biochar dose (2.5 g) performed better. Considering the compounds’ concentrations and the BC dose, BC from grapevine pruning residues demonstrated a potential use in the uptake of specific polyphenols from olive leaves, making it a promising adsorbent for such applications.

## 1. Introduction

The main role of agricultural production is to supply food to the ever-growing population, but at the same time, it has a negative impact on the environment, considering the use of pesticides and fertilizers, the emissions of various gases, and the production of biomass residues [1,2,3]. Biomass residues are usually rich in compounds that can be used for different purposes, such as in the food, chemical, and pharmaceutical industries [4,5]. 

The olive tree (*Olea europea* L.) is commonly grown for its fruit, which is used in olive oil production. According to Eurostat [6], the European Union counted 4.49 million ha under olive trees in 2017, mainly in the Mediterranean area, with Spain and Italy leading the production of the fruit. Olive pomace, olive mill wastewater, and branches and leaves are the main biomass residues from olive oil production and olive-tree growing. To achieve higher and healthier yields, pruning is performed, as an agro-technical measure, to remove the excess branches and leaves [7,8,9]. The production of olive leaves and branches from pruning has been estimated to be 25 kg per olive tree [10]. In most cases, the obtained biomass is improperly disposed of, incinerated or spread across the field [9], with a resulting series of negative environmental effects, such as emissions of greenhouse gases; air, water, and soil contamination; and waste of resources [11]. Olive biomass is considered a byproduct with no value [12], but different authors [13,14,15] have reported high polyphenolic concentrations in olive residues. Secoiridoids, in particular oleuropein and its derivatives, followed by hydroxytyrosol, are the main olive leaf components, a list which also includes other polyphenols and simple phenolics (caffeic acid, tyrosol), triterpenes, oleanolic acid, and flavonoids (i.e., rutin, apigenin, kaempferol, and luteolin) [16,17].

The concentrations of the extracted bioactive compounds depend on the olive variety and, mostly, on the extraction technique [18]. Different solvents are used in the extraction of bioactive compounds to achieve better performance, extraction, and yields [19]. Classic extraction procedures often involve the use of unsustainable solvents such as methanol, acetonitrile, or hexane [14]. To reduce the impact on the environment and to contribute to the fight against climate change, the European Commission adopted the European Green Deal strategy, including the Chemicals Strategy for Sustainability Towards a Toxic-Free Environment [20], with reducing human and environmental risks posed by certain hazardous chemicals as one of the strategy’s goals. 

The use of eco-friendly chemicals, as well as the development of green extraction technologies, are challenges for researchers. Many studies have reported the high extraction of bioactive compounds using green solvents such as ethanol [21], acetone [19], or natural deep eutectic solvents (NADES) [22]. However, water is the most naturally available solvent, covering more than 70% of the Earth’s surface. Because of its polarity, low supply costs, easy availability, and low environmental impact, water could be a potential solution in bioactive compounds extraction. To improve aqueous extraction and achieve better yields, different technologies are used nowadays, such as microwave-assisted extraction (MAE) or ultrasound-assisted extraction (UAE) [12]. When choosing the extraction technology, it is necessary to take into account various factors such as temperature, extraction time, and solid-to-liquid ratio [23].

Viticulture in the Mediterranean region represents 40% of the total area under grapevine production worldwide [24], generating huge amounts of residues due to agro-technical measures [25]. Pyrolysis is a common decomposition method for biomass under anaerobic conditions and in a temperature range of 300–900 °C [26], representing a potential solution for viticulture biomass valorization by its encouragement of the circular economy. The product of pyrolysis is biochar, described as a carbon rich, heterogeneous solid material characterized by a porous structure and low polarity [27,28]. It has a wide application in environmental remediation due to its strong adsorption capacity [26,27]. Biochar (BC) from lignin or cellulose-rich plants could form more porous structures [26], making it more suitable for the removal of compounds. Furthermore, due to its properties such as large surface area, the presence of negatively charged organic functional groups, and its cation exchange capacity, it is considered to be an efficient adsorbent [28,29,30]. Several authors have reported biochar’s capacity in the removal of pollutants and phenols [26,29,31]. 

The aim of this research was to evaluate water as a green solvent in the extraction of polyphenols from olive leaves and the potential of BC from grapevine pruning residues in the adsorption of bioactive compounds from olive leaf extracts. Using three different extraction techniques, maceration (MAC), MAE, and UAE, in addition to solid-to-liquid ratio (s/l) and temperature modulation, the polyphenolic profiles and antioxidant capacities of the obtained extracts were investigated. The BC produced from grapevine pruning residues was tested as an adsorbent for polyphenolic compounds from the obtained olive leaf extract using Langmuir and Freundlich isotherm models.

## 2. Materials and Methods

### 2.1. Plant Material

Olive leaves (*O. europea* L.) were collected in June 2022 (approximately 1 kg of leaves was collected in total) from an experimental olive plantation at the Institute of Agriculture and Tourism, Poreč, Croatia. The leaves were air-dried at 30 °C until they reached a constant weight (Memmert UF160, Schwabach, Germany), and ground in an ultra-centrifugal mill (Retsch ZM 200, Haan, Germany) to a 0.2 mm fine powder. 

### 2.2. BC Production

Residues from grapevine pruning destined for biochar (BC) production were gathered at the Institute of Agriculture and Tourism in Poreč, Croatia. These residues originated from an experimental vineyard featuring the ‘Istrian Malvasia’ cultivar (*Vitis vinifera* L.). The canes underwent pyrolysis using a Kon-Tiki system, with the peak temperature reaching 400 °C. The resulting biochar was subjected to air drying at a constant temperature of 30 °C (Memmert UF160, Schwabach, Germany), followed by grinding in a mortar mill (Retsch, RM 200, Haan, Germany). The powder was subsequently sifted through a test sieve to achieve particle sizes ranging from 125 to 250 µm. The details of the production process and the characterization of the produced biochar is reported in Prelac et al. [29].

### 2.3. The Extraction of Polyphenolic Compounds

To investigate the potential of water as a sole solvent for the extraction of polyphenolic compounds from olive leaves, an experiment with a balanced design was set up with s/l ratio and temperature as the main factors, with five levels each, and with three replications. The samples were fused with 25 mL of distilled water at 20 °C to obtain s/l ratios of 1:25, 1:50, 1:100, 1:250, and 1:500, using 1, 0.5, 0.25, 0.1, and 0.05 g of olive leaves, respectively. The investigated extraction temperatures were 30, 45, 60, 75, and 90 °C, achieved by either an ultrasonic bath (40 kHz, 300 W ultrasound power, 400 W heater power, MRC 250 H, Holon, Israel), a microwave unit with the power set at 800 W (Milestone Ethos Up, Sorisole, Italy), or a water bath (GFL 1013, Burgwede, Germany). Each extraction lasted for a period of 30 min, including a 5 min pre-heating phase, and the samples were then left to cool for 24 h. Following this, the extract underwent centrifugation at 16,000× *g* for 5 min using a Domel Centric 350 centrifuge (Železniki, Slovenia). The resulting supernatant was filtered through a 0.22 µm syringe filter, and the extracts were subsequently stored at −18 °C until analysis.

### 2.4. Analysis of Polyphenolic Compounds in Olive Leaf Extracts

The polyphenolics analysis was performed on a Shimadzu Nexera UPLC-PDA instrument consisting of two pumps (LC -40DX3, Shimadzu, Kyoto, Japan), a degasser (DGU-405, Shimadzu, Kyoto, Japan), a controller (SCL-40, Shimadzu, Kyoto, Japan), an autosampler (SIL-40CX3, Shimadzu, Kyoto, Japan), a diode array detector (SPD-M40, Shimadzu, Kyoto, Japan), and a heated column compartment (CTO-40C, Shimadzu, Kyoto, Japan). The column for separation of the analytes was a Poroshell 120 EC-C18 2.7 µm (2.1 mm × 150 mm) (Agilent, Palo Alto, CA, USA). The column temperature was set at 40 °C. A flow rate of 0.4 mL/min was established. The binary gradient elution of mobile phases A and B followed this sequence: 0–18 min, transitioning from 98% A to 40% A; 18–20 min, 40% A to 20% A; 20–21 min, 20% A to 2% A; 21–24 min, maintaining 2% A; 24–25 min, 2% A to 98% A; and 25–30 min, 98% A. Solvent A was water, while solvent B was methanol, both containing 0.2% acetic acid (*v/v*). The entire run’s duration was 30 min. The polyphenolics detected in olive leaf extracts included 11 compounds, but due to low concentrations, only hydroxytyrosol, luteolin-7-*O*-glucoside, apigenin-7-glucoside, and oleuropein were further investigated. Polyphenolic compounds were quantified using calibration curves obtained with serial standard dilutions of hydroxytyrosol (y = 28,440.06x − 4 455.44, R^2^ = 0.9999), apigenin-7-glucoside (y = 29,478.83x + 29 675.27, R^2^ = 0.9999), and oleuropein (y = 16,109.34x − 369.53, R^2^ = 0.9999). Luteolin-7-*O*-glucoside was quantified using luteolin-4-*O*-glucoside (y = 53,613.20x + 20,071.95, R^2^ = 0.9998). Quantification of hydroxytyrosol and oleuropein was performed at 280 nm, while apigenin-7-*O*-glucoside and luteolin-7-*O*-glucoside were quantified at 360 nm. The results were expressed in µg/g dry weight (DW) of olive leaves.

### 2.5. Antioxidant Capacity of Olive Leaf Extracts 

The assay for DPPH radical scavenging activity was conducted following the method outlined by Brand-Williams et al., with certain modifications [32]. The reaction mixture comprised 200 µL of freshly prepared 0.02M DPPH radical and 100 µL of the extract. The well-plate was placed in the dark at 25 °C for 30 min, and the antioxidant capacity values were measured at an absorbance of 517 nm (Tecan Infinite 200 Pro M Nano+, Männedorf, Switzerland) after the specified reaction time. The results were calculated against a Trolox calibration curve (ranging from 20 to 100 µM, y = −13.47x + 13.407; R^2^ = 0.9998) and expressed as µmol of Trolox equivalents per gram of dry weight (µmol TE/g DW).

For the ferric reducing antioxidant power assay (FRAP), the procedure followed that of Benzie and Strain, with some adjustments [33]. The reaction mixture consisted of 100 µL of extract and 200 µL of freshly prepared FRAP reagent. The well-plate was stored in the dark at 25 °C for 10 min, and absorbance was measured at 593 nm (Tecan Infinite 200 Pro M Nano+, Männedorf, Switzerland). FRAP values were calculated using a Trolox calibration curve (ranging from 20 to 100 µM; y = 6.82156x + 0.02291; R^2^ = 0.9999) and expressed as µmol TE/g DW.

The oxygen radical absorbance capacity (ORAC) was investigated according to the description of Ou et al., with slight modifications [34]. In summary, 37.5 µL of extracts were mixed with 225 µL of freshly prepared 4 µM fluorescein solution, and the reaction mixture was incubated at 37 °C for 30 min. Subsequently, 37.5 µL of freshly mixed AAPH was added to the incubated mixture. Excitation (485 nm) and emission (528 nm) wavelengths were measured for 120 min (Tecan Infinite 200 Pro M Nano+, Männedorf, Switzerland). ORAC values were calculated from a Trolox calibration curve (ranging from 4 to 20 µM; y = 0.0404x − 0.0005, R² = 0.9999) and expressed as µmol TE/g DW.

### 2.6. Adsorption Capacity of BC Derived from Grapevine Pruning Residues

Based on the experimental data, olive leaf extract obtained by UAE at 90 °C and an s/l ratio of 1:100 was used for further experiments with BC adsorption. The first adsorption experiment was carried out by fusing batches of 10 mg BC with olive leaf extract at concentrations ranging from 5 to 50 mg/L to determine the capacity of BC to adsorb polyphenols. In the second adsorption experiment, the effect of BC dosage on the adsorption of polyphenolic compounds was investigated by using BC at dosages of 0.5, 1.0, 1.5, 2.0, and 2.5 g/L, and fusing it with olive leaf extract at a concentration of 3 g/L.

The mixtures underwent rotation for a duration of 24 h at 25 °C using the Biosan Multi RS60 apparatus (Riga, Latvia). Afterward, the samples were filtered through a 0.22 μm nylon filter into an HPLC vial, and analyses were carried out on an LC-ESI-QqQ system from Shimadzu, in Kyoto, Japan. This instrumentation included a column oven compartment (Nexera CTO-40C), an autosampler (Nexera SIL-40CX3), two solvent delivery units (Nexera LC-40DX3), and a QqQ mass spectrometer (LCMS8045). To identify targeted compounds, specific ions and retention times were compared with analytical standards. The separation of these compounds was accomplished by injecting 1 µL of the extract onto a C18, 2.1 mm × 150 mm, 2.7 µm core-shell column from Advanced Materials Technology in Wilmington, DE, USA. The oven temperature was maintained at 40 °C. The binary gradient elution of mobile phases occurred as follows: 0 min to 1 min, 98% A; 1 min to 16 min, 98% A to 40% A; 16 min to 21 min, 40% A to 0% A; 21 min to 24 min, 0% A; 24 min to 25 min, 0% A to 98% A; and 25 min to 30 min, 98% A. In this gradient, mobile phase A consisted of water, and mobile phase B comprised methanol, both containing 0.1% acetic acid (*v*/*v*). The flow rate was set at 0.30 mL/min. The LC-ESI-QqQ analysis revealed 31 polyphenolic compounds in the olive leaf extract. After the adsorption on the BC, only the compounds with the highest concentrations were fitted with the isotherm models. The studied compounds were vanillic acid-4-glucoside, hydroxytyrosol, luteolin-7-*O*-glucoside, luteolin-7-*O*-rutinoside, apigenin-7-*O*-glucoside, and oleuropein. The results were expressed in µg/g DW.

The results of the first experiment were fitted with the Langmuir and Freundlich isotherms to better understand the adsorption dynamics of the polyphenolics on the surface of the grapevine-pruning-residue BC. The Langmuir isotherm is explained as follows [35]:

1/q_eL_ = 1/q_max_ + 1/(K_L_ × q_max_) × 1/γ_e_ (1)

where q_eL_ represents the amount of adsorbate concentration in the solid phase at equilibrium (mg/g), 1/q_max_ is the slope of linear equation, 1/(K_L_ × q_max_) is the y-intercept, K_L_ signifies the affinity constant (L/mg), q_max_ is the maximum monolayer adsorption capacity (mg/g), and γ_e_ is the amount of adsorbate concentration in the liquid phase at equilibrium (mg/L). The equation was plotted as 1/q_eL_ vs. 1/γ_e_, and the coefficient of determination (R^2^) was calculated. Additionally, the R_L_ factor was calculated to determine the favorability of Langmuir isotherms, as described below:

R_L_ = 1/(1 + K_L_ × γ_0_) (2)

where K_L_ is the affinity constant (L/mg), and γ_0_ is the initial concentration of the adsorbate (mg/L). 

The Freundlich isotherms were calculated using the equation below and plotted as log q_eF_ vs. log γ_e_. The Freundlich isotherm constant (K_F_/(mg/g) × (L/g)^n^), adsorption intensity (n), and R^2^ were calculated using the plot.

log q_eF_ = log K_F_ + 1/n × log γ_e_ (3)

### 2.7. Statistical Analysis

Statistical analysis of the data was conducted through Statistica 13.4 (Tibco, Inc., Palo Alto, CA, USA) using analysis of variance (ANOVA) and Tukey’s post hoc test, considering significant differences at a *p*-value of ≤0.05 for the comparison of group mean values. Furthermore, the graphs depicting the relationship between temperature and s/l ratio in the extracted data were generated using the Distance Weighted Least Square algorithm in Statistica 13.4 (Tibco, Inc., Palo Alto, CA, USA).

## 3. Results

### 3.1. The Influences of Extraction Method, Temperature, and Solid-to-Liquid Ratio on Olive Leaves’ Polyphenolic Compound Content and Antioxidant Capacity

The polyphenols detected in olive leaf extracts included 11 compounds, but due to low concentrations, only hydroxytyrosol, luteolin-7-*O*-glucoside, apigenin-7-glucoside, oleuropein, and their sum were further analyzed. 

**Figure 1:**
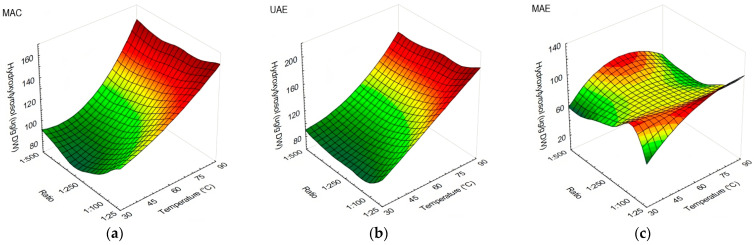
Response surface plots showing the influence of temperature against s/l ratio in olive leaf extracts on the content of hydroxytyrosol (µg/g DW): (**a**) extraction performed using MAC; (**b**) extraction performed using UAE; (**c**) extraction performed using MAE.

**Figure 2:**
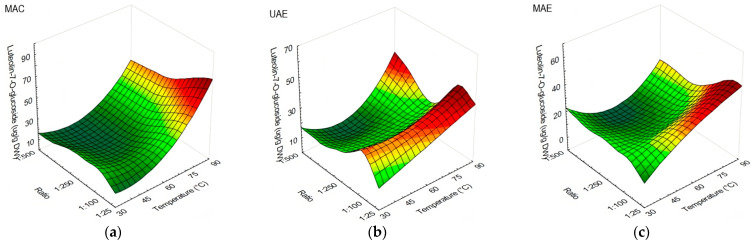
Response surface plots showing the influence of temperature against s/l ratio in olive leaf extracts on the content of luteolin-7-*O*-glucoside (µg/g DW): (**a**) extraction performed using MAC; (**b**) extraction performed using UAE; (**c**) extraction performed using MAE.

**Figure 3:**
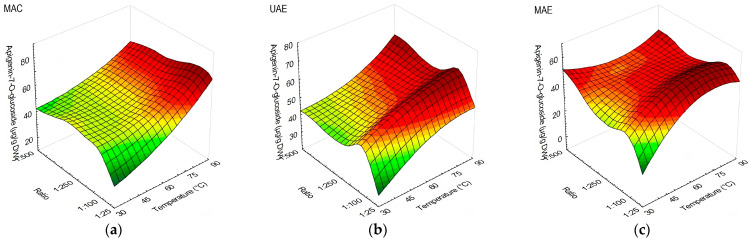
Response surface plots showing the influence of temperature against s/l ratio in olive leaf extracts on the content of apigenin-7-*O*-glucoside (µg/g DW): (**a**) extraction performed using MAC; (**b**) extraction performed using UAE; (**c**) extraction performed using MAE.

The effects of temperature against s/l ratio on polyphenolic profile while using MAC, UAE, and MAE are shown in Figure 1, Figure 2 and Figure 3. In general, all investigated compounds yielded better at 75 °C or above. Higher yields of hydroxytyrosol were obtained at extraction temperatures of 75 and 90 °C, with no influence of s/l ratio when MAC (Figure 1a) and UAE (Figure 1b) were applied. On the contrary, MAE (Figure 1c) yielded better at all temperatures used, with visible s/l ratio influence; s/l ratios of 1:25 and 1:50 were more favorable when temperatures from 45 to 90 °C were used. The s/l ratios from 1:50 to 1:100 yielded better at 30 °C, while 1:25 achieved the lowest yield at the same temperature.

As for luteolin-7-*O*-glucoside, there was an evident effect of s/l ratio and temperature on the extraction yield. The highest yield of luteolin-7-*O*-glucoside was detected at 90 °C when applying MAC with s/l ratio from 1:25 to 1:250, while the lowest yield was obtained using s/l ratio 1:500 at 45, 75, and 90 °C (Figure 2a). In UAE application, s/l ratio 1:100 performed better in all temperatures used, while s/l ratio 1:500 obtained the lowest amount of luteolin-7-*O*-glucoside (Figure 2b), except when the temperature was set at 75 or 90 °C. When applying MAE at a temperature of 30 °C, the influence of ratio on the yield of the investigated compound was visible (Figure 2c). Specifically, as the s/l ratio decreased, the concentration of the investigated compound increased. The opposite trend was observed at 60 °C; the concentration of the compound increased as the ratio increased. Furthermore, at temperatures of 45, 75, and 90 °C, an increase in yield was recorded at ratios of 1:50 and 1:100; when reducing the ratio, the yield would also decrease.

The yield of apigenin-7-*O*-glucoside was affected by temperature in MAC application, performing better at 90 °C with no visible s/l ratio influence (Figure 3a). As for UAE, comparable results were obtained from 60 to 90 °C, and with ratios starting from 1:50 or less (Figure 3b). MAE obtained the lowest amount of apigenin-7-*O*-glucoside when utilizing 30 °C and an s/l ratio of 1:25, in comparison with other methods, while the highest yields were observed at 45 °C and with s/l ratios of 1:100 and 1:250, 60 °C and s/l ratio 1:25, and 75 and 90 °C with s/l ratios of 1:50 and 1:100 (Figure 3c).

**Figure 4:**
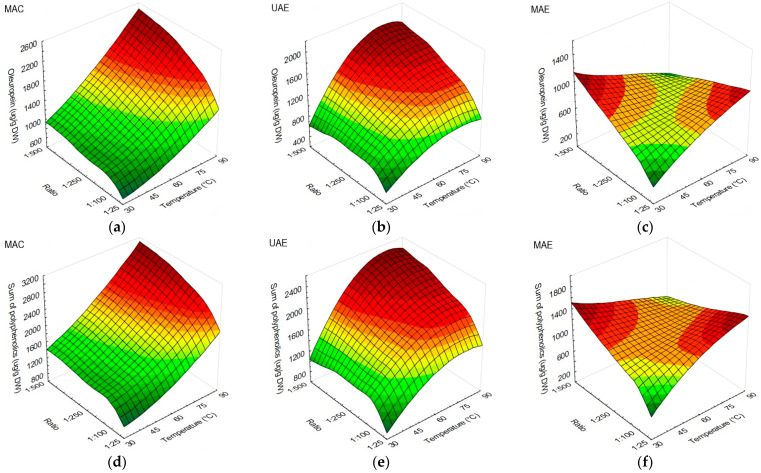
Response surface plots showing the influence of temperature against s/l ratio in olive leaf extracts on the content of: (**a**) oleuropein (µg/g DW) using MAC; (**b**) oleuropein (µg/g DW) using UAE; (**c**) oleuropein (µg/g DW) using MAE; (**d**) sum of polyphenolics (µg/g DW) using MAC; (**e**) sum of polyphenolics (µg/g DW) using UAE; (**f**) sum of polyphenolics (µg/g DW) using MAE.

Oleuropein was the compound found most abundantly in olive leaves, and accordingly, the sum of polyphenolics resulted in a similar trend. MAC and UAE obtained higher yields in similar conditions, with visible temperature and s/l ratio influences on the yield. MAC performed better at 75 and 90 °C when s/l ratios of 1:250 or 1:500 were used (Figure 4a,d), while in the application of UAE, similar results were obtained at temperatures of 60, 75, and 90 °C (Figure 4b,e). MAE has obtained higher yields of oleuropein and polyphenolics at 30 °C, with results comparable to those at 75 and 90 °C (Figure 4c,f). Overall, MAC has extracted the highest yield of total polyphenolics (3037 ± 22 µg/g DW) at 90 °C and an s/l ratio of 1:500, followed by UAE using an s/l ratio of 1:500 at 60 °C (2476 ± 22 µg/g DW), 75 °C (2383 ± 29 µg/g DW), and 90 °C (2405 ± 22 µg/g DW). As for MAE, the highest yield was observed at 30 °C and with a 1:500 s/l ratio (1582 ± 122 µg/g DW). 

The results for the antioxidant capacity followed the polyphenolics profiles results. The results are available in Supplementary Table S1. The results have shown the influences of s/l ratio and temperature in ORAC assays when MAC and UAE were applied. MAC results have shown an effect of temperature and s/l ratio at temperatures from 30 to 60 °C. The highest yield was obtained at 90 °C and with an s/l ratio of 1:100. In the case of UAE, there was an evident influence of those two parameters; the yield was increasing as the temperature increased and ratio decreased. MAE resulted in comparable values within the group, obtaining a higher yield at 75 °C and an s/l ratio of 1:100, and a lower yield at 30 °C and with an s/l ratio of 1:25. DPPH assay results have shown the influence of temperature when MAC was applied, yielding better at 75 and 90 °C. On the contrary, UAE yielded better at temperature range from 45 to 60 °C, while MAE obtained higher values at 45 and 75 °C, regardless of the s/l ratio. FRAP assay results suggested the use of MAC at 90 °C using an s/l ratio of 1:25, and UAE at 75 °C with the same ratio. As for MAE, the results were comparable within the group, but indicated obtaining higher yields at 75–90 °C and an s/l ratio of 1:25.

### 3.2. Adsorption Capacity

#### 3.2.1. Langmuir and Freundlich Isotherms

**Figure 5:**
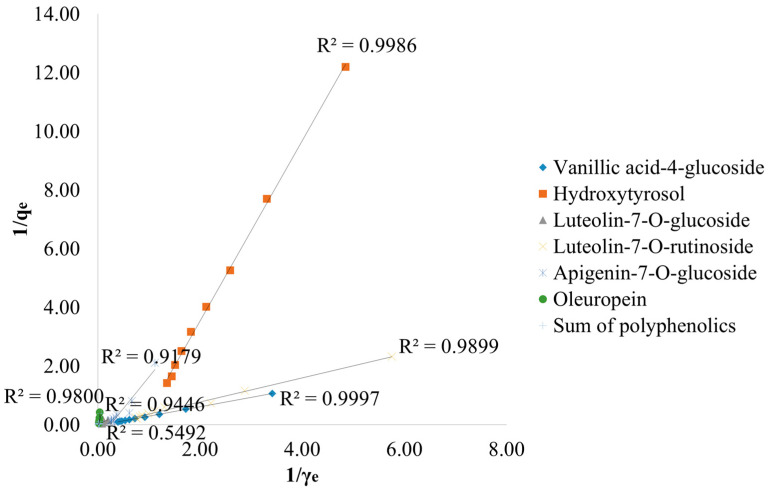
Langmuir adsorption isotherms for the adsorption of specific compounds by biochar are expressed in terms of key parameters, among which R^2^ represents the coefficient of determination, q_e_ signifies the amount of adsorbate concentration in the solid phase at equilibrium (mg/g), and γ_e_ denotes the amount of adsorbate concentration in the liquid phase at equilibrium (mg/L).

**Figure 6:**
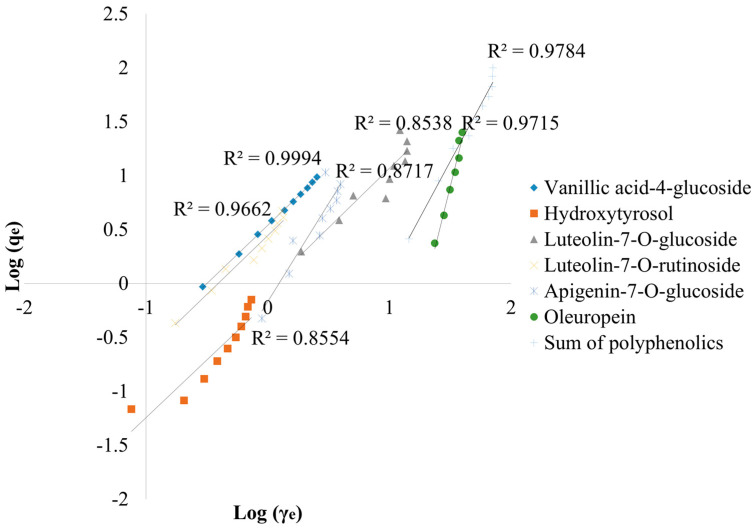
The Freundlich adsorption isotherms for the adsorption of specific compounds by biochar are characterized by key parameters, among which R^2^ represents the coefficient of determination, q_e_ indicates the amount of adsorbate concentration in the solid phase at equilibrium (mg/g), and γ_e_ signifies the amount of adsorbate concentration in the liquid phase at equilibrium (mg/L).

The adsorption results of polyphenolic compounds from the first experiment were fitted with the Langmuir and Freundlich isotherm models in order to understand and determine the process of the adsorption of the targeted compounds onto BC. Figure 5 shows the Langmuir isotherm models for vanillic acid-4-glucoside, hydroxytyrosol, luteolin-7-*O*-glucoside, luteolin-7 *O*-rutinoside, apigenin-7-*O*-glucoside, and oleuropein, as well as the sum of polyphenols, with the R^2^ coefficients ranging from 0.549 to 0.999. Freundlich isotherm models are shown in Figure 6, where the R^2^ ranges from 0.854 to 0.999. 

As shown in Table 1, all investigated compounds obtained R^2^ coefficients higher than 0.90, suggesting the suitability of the Langmuir model for the above-mentioned compounds’ adsorption, with the exception of luteolin-7-*O*-glucoside, for which the R^2^ was 0.5492. However, the other obtained parameters should also be considered in the adsorption model suitability determination. The Langmuir model was not suitable for vanillic acid-4-glucoside, luteolin-7-*O*-rutinoside, apigenin-7-*O*-glucoside, and oleuropein due to negative K_L_ and R_L_ values. Furthermore, the Langmuir model fitted better only in hydroxytyrosol and total polyphenolics adsorption. Total adsorption of polyphenols obtained a twofold-higher maximum monolayer adsorption capacity (q_max_), followed by hydroxytyrosol and the highest affinity constant (K_L_), respectively. The Freundlich model was more favorable for all investigated compounds, resulting in R^2^ ≥ 0.854. The highest R^2^ coefficient was obtained in vanillic acid-4-glucoside adsorption (0.999), followed by total polyphenols (0.978) and oleuropein (0.972). The highest adsorption capacity (K_f_) was reached with oleuropein (20.4 (mg/g) × (L/g)^n^). The 1/n values were in a range between 0 and 1 for all investigated compounds.

#### 3.2.2. Effects of BC Dosages in Polyphenolic Adsorption

**Figure 7:**
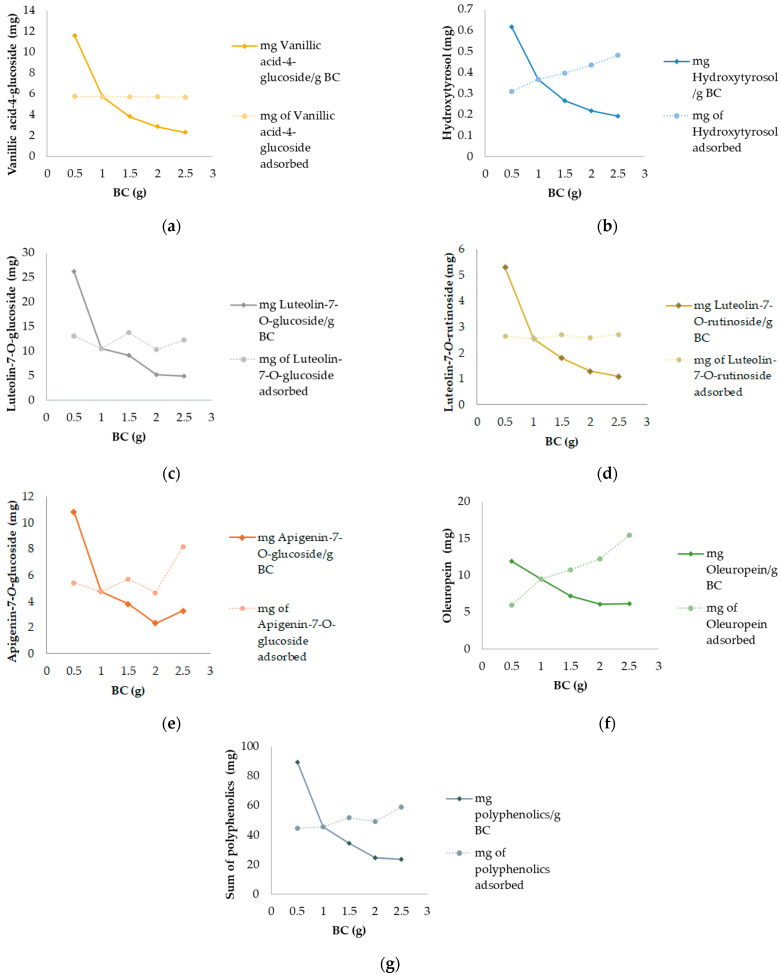
Influences of different biochar (BC) dosages in adsorption of polyphenolic compounds from olive leaf extracts with 24 h contact: (**a**) vanillic acid-4-glucoside; (**b**) hydroxytyrosol; (**c**) luteolin-7-*O*-glucoside; (**d**) luteolin-7-*O*-rutinoside; (**e**) apigenin-7-*O*-glucoside; (**f**) oleuropein; (**g**) sum of polyphenolics.

Figure 7 illustrates the outcomes of the experiment conducted with various biochar (BC) dosages while maintaining a consistent concentration of olive leaf extract (3 g/L). The results are presented in terms of milligrams of the targeted compound adsorbed per gram of BC and the overall amount of adsorbed compound under different BC dosages. Notably, the 0.5 g BC dosage exhibited the highest adsorption for all compounds, demonstrating a diminishing trend as the BC quantity increased. In the total adsorption capacity experiment, the influence of BC dosages on adsorption was evident after reaching equilibrium for all investigated compounds, excluding vanillic acid-4-glucoside and luteolin-7-*O*-rutinoside.

Specifically, the maximum amount adsorbed per gram of BC for vanillic acid-4-glucoside was observed at the 0.5 g BC dosage (11.6 mg vanillic acid-4-glucoside/g BC), gradually decreasing to 2.28 mg vanillic acid-4-glucoside/g BC with an increase in BC dosage (Figure 7a). However, the total amount of vanillic acid-4-glucoside adsorbed remained consistent, ranging from 5.71 to 5.78 mg, indicating no significant impact of BC dosages on overall adsorption (Figure 7a).

As shown in Figure 7b, the highest amount of hydroxytyrosol adsorbed per g of BC was observed when 0.5 g of BC was used (0.62 mg hydroxytyrosol/g BC), decreasing to 0.19 mg hydroxytyrosol/g BC as the BC dosage increased, while the total amount of hydroxytyrosol adsorbed increased as the BC dosage did, ranging from 0.31 mg at 0.5 g of BC to 0.48 mg of hydroxytyrosol when 2.5 g of BC was applied. 

Luteolin-7-*O*-glucoside adsorption results per g of BC were the highest among other individual compounds, ranging from 26.2 to 4.94 mg (Figure 7c). The total adsorption values obtained a a s/l zag pattern, suggesting the influence of BC dosage on adsorption; the highest amounts of luteolin-7-*O*-glucoside were adsorbed using 0.5 (13.1 mg), 1.5 (13.8 mg), and 2.5 g (12.4 mg) of BC, as shown in Figure 7c. 

As for luteolin-7-*O*-rutinoside, the highest amount adsorbed per g of BC was obtained with the application of 0.5 g of BC (5.29 mg luteolin-7-*O*-rutinoside/g of BC), with adsorption per g of BC decreasing as the dosage increased (Figure 7d). The total adsorption capacity demonstrated no influence on luteolin-7-*O*-rutinoside adsorption when different BC dosages were applied, with values ranging from 2.65 to 2.72 mg (Figure 7d).

Apigenin-7-*O*-glucoside results followed the trends of luetolin-7-*O*-glucoside, obtaining the highest adsorption when 0.5 g of BC was applied (10.9 mg apigenin-7-*O*-glucoside/g BC) and showing a decreasing trend as the BC dosage increased, as well as a similar zig-zag pattern in the total-adsorption-capacity experiment. The dosage of 2.5 g of BC adsorbed the highest amount of apigenin-7-*O*-glucoside (8.18 mg), as shown in Figure 7e. 

Oleuropein values for the adsorption experiment per g of BC resulted in highest adsorption when 0.5 g of BC was applied (11.9 mg oleuropein/g BC), with decreases in the adsorbed amount of the compound as the dosage of BC increased. The total adsorption values obtained a linear increasing trend, adsorbing 5.95 mg of oleuropein with 0.5 g BC dosage, with an increase to 15.5 mg when 2.5 g of BC was applied, suggesting an evident effect of BC dosage on the adsorption of polyphenols. Both results are shown in Figure 7f.

In Figure 7g, the results for the sum of polyphenols are shown. The BC dosage of 0.5 g followed the previous adsorption compounds trend by adsorbing the highest amount of total polyphenols (89.4 mg/g BC), with a decrease in the amount of adsorbed compound to 23.6 mg/g BC when 2.5 g of BC was applied. The total adsorption experiment obtained a linear relationship between the adsorbed amount of polyphenols and BC dosage, ranging from 44.7 mg of polyphenols when 0.5 g of BC was used, to, after a slight increase at 2.5 g of BC, 58.9 mg of polyphenols.

## 4. Discussion

In this study, olive leaves were used as a source material rich in bioactive compounds, water as a solvent for extractions, and BC from grapevine pruning residues as an adsorbent, in order to utilize agricultural-production and food biomass and wastes, leading to a sustainable production, and obtain an eco-friendly approach in extraction and adsorption processes. 

The extraction of natural compounds is a demanding process in which a non-toxic solvent should be used, especially if it is carried out for food purposes [36,37]. Water is considered to be one of the greenest solvents used in extraction processes. The number of experiments in which water was used as an environmentally friendly solvent has increased [38]. Benincasa et al. investigated the effects of different water types in the extraction of high-valued compounds from olive leaves, stating that ultrapure water stimulates the migration process of oleuropein from the leaves to the solution more than micro-filtered or osmosis-treated water [14]. In our previous work, detailed observation of water as a green solvent was given, and the study reported the efficiency of its use as a solvent in the extraction of polyphenols from onion peels [39]. In order to extract a higher amount of polyphenols from olive leaves, different techniques are usually applied, with regard given to the extraction temperature and solvent as important parameters for the recovery of phenols [37]. 

In this work, three different methods were used (MAC, UAE, and MAE), with different temperature and ratio levels investigated. Five temperatures (30, 45, 60, 75, and 90 °C) and five s/l ratios (1:25, 1:50, 1:100, 1:250, and 1:500) were examined, with the aim being to reduce the energy consumption and obtain a high yield of bioactive compounds by reducing the use of solvents and increasing the use of biomass. As the results suggested, the highest yields of polyphenols and highest antioxidant activity were obtained at temperatures of 75 or 90 °C, regardless of the extraction method, probably due to cell-wall disruption caused by high temperatures [40]. Khemakhem et al. have reported an improvement in total phenolic content, antioxidant activity, and oleuropein amounts by increasing the temperature from 10 to 70 °C in olive leaf extractions, using the conventional extraction method and UAE [41]. Furthermore, in another research effort [42], different temperature ranges, times and s/l ratios in conventional aqueous olive leaf extractions were investigated. The optimal conditions for the highest yield of phenolic compounds were found to be 90 °C for 70 min, and at an s/l ratio of 1:100. To minimize the use of solvent, the authors suggested the use of an s/l ratio of 1:60 for obtaining 80% of the total phenolic compounds and maximized antioxidant capacity. A similar trend was observed in this study in the antioxidant activity assays: the highest yield in ORAC was obtained at 90 °C and an s/l ratio of 1:100 for MAC and UAE, while MAE obtained better results at 75 °C. UAE and MAE performed similarly in the DPPH assay, with better yields at temperatures from 45 to 60 °C, and 45 to 75 °C, respectively. In MAC, higher temperatures were needed, ranging from 75 to 90 °C. Finally, FRAP results suggested the use of a temperature range from 75 to 90 °C and an s/l ratio of 1:25 to achieve higher values. According to the results, high temperature is not a limiting factor in the extraction of polyphenols from olive leaves, considering the obtained high yield and preserved antioxidant capacity.

However, when observing the individual compounds and their yields, the influences of the extraction method itself, temperature, and ratio were noticeable. In hydroxytyrosol extraction, an evident influence of temperature was noticeable in MAC and UAE applications. The highest yield was obtained when 75 or 90 °C were used. As for MAE, an s/l ratio effect was observed, yielding better at 45 to 90 °C and using ratios of 1:25 or 1:50. Luteolin-7-*O*-glucoside and apigenin-7-glucoside were extracted in higher amounts at temperatures from 45 to 90 °C when MAE was used, as well as when MAC was used at 90 °C with ratios from 1:25 to 1:250. As for UAE in luteolin-7-*O*-glucoside extraction, the highest yield was achieved using an s/l ratio of 1:100 at all investigated temperatures, suggesting the possibility of reducing energy consumption if lower temperatures are applied, while temperatures from 60 to 90 °C were more favorable in apigenin-7-*O*-glucoside extraction. The main compound found in olive leaves was oleuropein, which is in accordance with previous results [43,44,45,46]. Accordingly, the sum of polyphenols performed similarly to oleuropein. The highest yield was obtained when using MAC or UAE at temperatures between 60 and 90 °C and with an s/l ratio of 1:250 to 1:500, while MAE performed better at 30 °C, providing an eco-friendly extraction due to lower energy consumption. According to Goldsmith et al., this phenomenon agrees with mass transfer principles: the gradient concentration is higher when there is more solvent present, leading to higher diffusion rates [42]. The amounts of the extracted compounds in this study were lower in comparison with other papers where 80%-methanol [43], ethanol: water (1:1) [47] or distilled water [48] were used. This phenomenon could be explained by the olive leaves’ collecting period having caused a decrease in the amount of polyphenols in a certain growing period [47,48,49,50]. Furthermore, the phenolic profiles and concentrations, as well as the antioxidant activity, are influenced by many factors, like seasoning age, climatic conditions, geographical position, cultivar, genetic factors [43,51], and exposure to low air temperatures [52]. Likewise, extraction conditions such as temperature, contact time, solvent, s/l ratio, and sample preparation also influence the polyphenolic content of the extract [17,53]. However, in the study conducted by Benincasa et al. with different water types, the amount of extracted oleuropein was almost threefold lower when compared with results in this study, while the concentration of hydroxytyrosol was fourfold higher in 24 h contact time [14]. The amount of hydroxytyrosol could increase during maturation or treatment due to oleuropein degradation [37]. This data indicates the appropriateness of the methods studied in this work for the aqueous extraction and preservation of higher concentrations of oleuropein.

Although in several works [37,54,55], innovative extraction methods such as MAE and UAE performed better than conventional methods, in this research, among all studied extraction techniques, MAE obtained the lowest yield of the investigated compounds. For instance, in this work, MAC has extracted a twofold higher amount of polyphenols at conditions of 90 °C and an s/l ratio of 1:500 if compared to the highest yield obtained by MAE (30 °C, s/l ratio of 1:500). In the research of da Rosa et al., MAC, UAE, and MAE were compared as to the recovery of bioactive compounds from Brazilian olive leaves [12]. The authors concluded that MAE was more efficient than the other two investigated methods. Valinger et al. also compared the efficiency of three methods of bioactive compounds’ extraction from olive leaves using conventional extraction, MAE, and microwave–ultrasound-assisted extraction, using water as a solvent and with an s/l ratio of 1:50 [56]. The combination of microwaves and ultrasounds obtained the best results when a temperature of 60 °C was used, while MAE performed better at 80 °C. Furthermore, Vizzarri et al. obtained important results in polyphenolic extraction from olive leaves in applying UAE by mixing 1.5 kg of dried leaves in 3 L of distilled water [57]. The results were almost 10-fold higher than in this work, suggesting a higher s/l ratio for a better extraction. However, in Lama-Muñoz et al. the conventional Soxhlet extraction method showed better performance in most of the variables analyzed from olive leaves when compared to the innovative pressurized liquid extraction [18]. Sánchez-Gutiérrez et al. obtained higher phenolic yield and antioxidant activity in conventional extraction as compared to MAE [58]. Finally, conventional extraction such as MAC is considered more suitable for extracting polar compounds such as oleuropein derivatives, apigenin-rutinoside, and luteolin-glucosides [52], a determination which was confirmed in this study.

Adsorption is a process in which soluble substances in the liquid phase transfer onto the surface or onto the bulk of the solid adsorbent [59], and it occurs by the formation of the physical or chemical bonds between a porous solid medium and a mixture of liquid or gas multi-component fluid [60]. The adsorption isotherm models describe the interaction mechanisms between the adsorbent and the adsorbate at constant temperature [60]. The Langmuir and Freundlich isotherm models are the most common and widely used. In this study, the mentioned models were used to determine the adsorption process by which polyphenols from olive leaf extracts adsorb onto the BC from grapevine pruning residues. BC properties and adsorption capacity were described in our previous works [29,39]. 

Among all investigated compounds, hydroxytyrosol and the total amount of polyphenols fitted better in the Langmuir model, obtaining the R^2^ closest to 1, suggesting the suitability of this model for the mentioned compounds’ adsorption. Hydroxytyrosol and total polyphenols obtained the highest values for maximum monolayer adsorption capacity, showing that the adsorption of those compounds was more favorable compared to the others, and suggesting a main monolayer adsorption reaction. Luteolin-7-*O*-glucoside has obtained an R^2^ of 0.5492, with the highest K_L_ indicating a strong interaction between the adsorbent and the adsorbate. The obtained value was higher than in the study by Aliakbarian et al., in which commercial activated carbon (AC; Sigma-Aldrich Chemical Co.) was used in phenolic adsorption from olive mill wastewater [61]. Vanillic acid-4-glucoside, luteolin-7-*O*-rutinoside, apigenin-7-*O*-glucoside, and oleuropein obtained an R^2^ coefficient equal to or higher than 0.9179. However, those compounds achieved negative K_L_ and R_L_ values. A negative K_L_ value was also obtained in our previous work with onion peel extracts [39]. The negative K_L_ value indicates that at high addition of adsorbent mass, adsorption does not follow Langmuir premises and the adsorption capacity reaches a specific limit by increasing the adsorbent mass at a certain point [62]. Moreover, the shape of the isotherm depends on the R_L_ value. The isotherm is unfavorable (R_L_ > 1), linear (R_L_ = 1), favorable (0 < R_L_ < 1), or reversible (R_L_ = 0) [63]. In this work, the value of R_L_ for vanillic acid-4-glucoside, luteolin-7-*O*-rutinoside, and apigenin-7-*O*-glucoside was negative, suggesting the inappropriateness of this model for the mentioned compounds’ adsorption. As for oleuropein and luteolin-7-*O*-glucoside, the values ranged from 0.00 to 1.32, and 0.00 to 0.91, indicating a reversible and unfavorable adsorption and a reversible and favorable adsorption, respectively. Nonetheless, in all of the other cases it was between 0 and 1, indicating that hydroxytyrosol and total polyphenols adsorption by BC is favorable. 

The Freundlich isotherm model was the best-fit isotherm for adsorption for all investigated compounds, ranging the values of the determination coefficient between 0.8538 and 0.9994. Those findings concur with Abid et al. [64] and Göktepeli et al. [65]. The highest R^2^ value was obtained by vanillic acid-4-glucoside, and the highest K_f_ value was achieved by oleuropein. This constant is used to describe the adsorption of solute molecules onto the surface of a solid adsorbent [66], suggesting a greater adsorption of oleuropein. The 1/n value was in range between 0 and 1, which indicates that the adsorption capacity increases with concentration, but at a decreasing rate [67], although representing favorable adsorption conditions [68]. Among all investigated compounds, hydroxytyrosol and total polyphenolics adsorptions fitted better with both Langmuir and Freundlich models. This observation was reported by Hadrich et al., who concluded that both the Langmuir and the Freundlich isotherm models fitted the equilibrium data of the adsorption process of hydroxytyrosol from olive leaves using a modified spherical activated carbon [69]. Those findings suggested that monolayer and heterolayer phenolic compounds may exist on the adsorbent surface due to the heterogeneity of the BC’s surface [64]. In our previous work [29], BC from grapevine pruning residues was investigated as an adsorbent for polyphenols. In that paper, the standard of oleuropein, among others, was used. The obtained results fitted in both Langmuir and Freundlich models, reaching high adsorption capacity and efficient adsorption by BC, while in this research, oleuropein fitted better with the Freundlich model. These differences between the results may be explained by the concentration of the compound in the solution, and the competition and interaction between the compounds in the extracts. The investigated compounds in this study range from weak to high polar: oleuropein, hydroxytyrosol, vanillic acid-4-glucoside, luteolin-7-*O*-glucoside, apigenin-7-*O*-glucoside, and the most polar compound, luteolin-7-*O*-rutinoside. Considering this data, as well as the adsorption results according to Freundlich’s model, the influence of the polarity of the compounds may affect the adsorption potential. Vanillic acid-4-glucoside and luteolin-7-*O*-rutinoside have obtained the lowest K_f_ values among all investigated compounds. Vanillic acid-4-glucoside and luteolin-7-*O*-rutinoside are more hydrophilic compared to oleuropein, which has obtained the highest K_f_ value. Furthermore, the structure of oleuropein is more complex and contains many glucose moieties. When compared to vanillic acid-4-*O*-glucoside and luteolin-7-*O*-rutinoside, its overall polarity may be reduced by the presence of more extended sugar units and higher complexity. In comparison to the other two compounds, oleuropein may exhibit better interactions with the polar functional groups on the surface of BC.

In regard to the effect of BC dosage on adsorption, the lowest BC dosage of 0.5 g performed the best adsorption capacity per g of BC, which rapidly decreased with increasing dosages, suggesting that compounds in the solution reached adsorption equilibrium. A similar observation was provided by Senol et al. [70] using commercial active carbon and Fseha et al. [71] in the removal of phenols from contaminated municipal wastewater using date palm frond BC. As for the absolute adsorption capacity, there was no visible influence of the dosage on vanillic acid-4-glucoside and luteolin-7-*O*-rutinoside, as previously reported [39]. Luteolin-7-*O*-glucoside and apigenin-7-*O*-glucoside performed similarly, obtaining a zig-zag pattern. However, if the lowest BC dosage, 0.5 g, was compared to the highest (2.5 g), the results would suggest a visible increase in adsorption. The greatest increase of an adsorbed compound was visible in oleuropein; the amount of the compound increased almost threefold, as the BC dosage did. A similar trend was observed in hydroxytyrosol and total polyphenols adsorption. The components that are found in the highest concentrations include oleuropein, hydroxytyrosol, and therefore the total amount of polyphenols, suggesting the influence of the compound concentration in the solution on the adsorption. In addition, it could also be explained by the formation of more adsorption sites as a consequence of the increased surface area [64], but could also be attributed to the nature of the adsorbate, specifically, its functional groups, polarity, molecular weight, and size [72].

There are some limitations to this study. The temperature of the pyrolysis process during the conversion of grape pruning residues could not be controlled, due to the design of the kon-tiki system. This could lead to lower active surface area and subsequently lower adsorption capacity of the investigated biochar. Another limitation of the study is the variation in the phytochemical content of the olive leaves, which is dependent upon genetic and environmental factors, and ultimately has an effect on the reported profile or adsorption kinetics on the biochar’s surface. Finally, olive leaf extracts have an abundance of hydrophilic compounds besides polyphenols, which can interfere with the adsorption process and also modify the adsorption kinetics.

## 5. Conclusions

Olive leaves are rich in valuable phytochemicals with high antioxidant activity. On the other hand, the intensive viticulture practiced today requires the implementation of certain measures in order to achieve a higher yield with better quality. In this paper, these two materials were investigated with the aim of biomass valorization in the agricultural sector. In order to extract bioactive compounds, water was used as a green solvent in three extraction methods, together with modulation of temperature and s/l ratio. The conventional MAC method proved to be more effective compared to the other methods at temperatures between 75 and 90 °C, obtaining higher polyphenol yields, especially oleuropein, as the main compound, with no adverse effects on antioxidant activity. Furthermore, using the Langmuir and Freundlich isotherm models, the potential of BC obtained from grapevine pruning residues in the adsorption of the polyphenolic compounds was tested. The Freundlich isotherm fitted better with all investigated compounds. Hence, a novel approach for a sustainable extraction of polyphenols from olive leaves, fitting with the principles of green chemistry and leading to eco-friendly processes of adsorption, was proposed in this study. The findings suggest the possibility of using water as solvent to obtain non-toxic and environmentally friendly extracts from olive leaves which can be used in the food industry or in other industries. The process could be easily implemented in the industry and has the potential to become an additional source of income for olive and grapevine growers.

## Figures and Tables

**Table 1:** Parameters of Langmuir and Freundlich models for targeted olive-leaf polyphenolic compounds adsorbed by grapevine-pruning-residues biochar.

	Parameters	VA-4-g	Hyty	L-7-g	L-7-r	A-7-g	Olp	Sum
Langmuir isotherm	q_max_ (mg/g)	1.54	3.08	1.21	2.44	0.47	0.05	6.83
	K_L_ (L/mg)	−0.28	0.38	102	−10.1	−4.17	−41.20	9.64
	R_L_	(−2.62)–(−0.01)	0.05–0.35	0.00–0.91	(−105)–(−0.02)	(−0.66)–(−0.05)	0.00–1.32	0.02–0.50
	R^2^	0.999	0.999	0.549	0.990	0.918	0.980	0.945
Freundlich isotherm	K_f_ (mg/g) × (L/g)^n^	0.32	1.20	1.44	0.40	1.35	20.4	9.49
	1/n	0.93	0.80	0.78	0.89	0.48	0.21	0.47
	R^2^	0.999	0.855	0.854	0.966	0.872	0.972	0.978

VA-4-g—Vanillic acid-4-glucoside; Hyty—Hydroxytyrosol; L-7-g—Luteolin-7-*O*-glucoside; L-7-r—Luteolin-7-*O*-rutinoside; A-7-g—Apigenin-7-*O*-glucoside; Olp—Oleuropein; Sum—Sum of polyphenols.

## Data Availability

All data is available within this article.

## Supplementary Materials

The following supporting information can be downloaded at: https://www.mdpi.com/article/10.3390/antiox13010001/s1, Table S1: Olive leaves polyphenolic compounds content (µg/g DW) using MAC, UAE and MAE at different extraction conditions.
