# Supplementary material for: Valorization of Olive Leaf Polyphenols by Green Extraction and Selective Adsorption on Biochar Derived from Grapevine Pruning Residues
Source: Antioxidants (Basel). 2023 Dec 19;13(1):1. doi: 10.3390/antiox13010001 (PMC10812658; doi:10.3390/antiox13010001)
Supplement: Supplementary file 1 [file antioxidants-13-00001-s001.zip › antioxidants-2739430-supplementary.pdf]

Supplementary table S1. Olive leaves polyphenolic compounds content (μg/g DW) using MAC, UAE and MAE at different extraction conditions.

Compound	Condition		Method			Method x temperature x ratio (p – value)
	**Temperature (°C)**	**Solid/liquid ratio**	**MAC**	**UAE**	**MAE**	
Hydroxytyrosol	30	1:25	103 ± 0 kl	113 ± 1 gi	7.1 ± 0.5 i	***
		1:50	117 ± 4 g-i	78.3 ± 5.8 i	108 ± 5 ab	
		1:100	93.4 ± 2.9 lm	82.2 ± 0.4 i	109 ± 7 ab	
		1:250	79.6 ± 1.7 n	85 ± 1.5 i	66.8 ± 14.7 gh	
		1:500	90.2 ± 1.2 m	87.3 ± 1.2 i	51.1 ± 5 h	
	45	1:25	113 ± 1 ij	123 ± 1 fg	96.1 ± 0.1 a-e	
		1:50	117 ± 1 g-i	124 ± 2 ef	109 ± 0 ab	
		1:100	105 ± 2 jk	107 ± 1 h	94.2 ± 0.4 a-e	
		1:250	90.1 ± 0.4 m	87.9 ± 0.6 i	89.1 ± 2.5 b-g	
		1:500	93.2 ± 5.2 lm	107 ± 3 h	101 ± 4 a-d	
	60	1:25	127 ± 0 fg	134 ± 0 de	102 ± 3 a-d	
		1:50	129 ± 1 ef	137 ± 1 de	87.4 ± 0.2 b-g	
		1:100	121 ± 3 f-i	125 ± 1 ef	74.3 ± 2.1 e-h	
		1:250	103 ± 0 kl	109 ± 3 h	82.1 ± 1.5 c-g	
		1:500	101 ± 1 kl	110 ± 2 h	91.7 ± 3.2 a-f	
	75	1:25	143 ± 2 cd	185 ± 1 ab	114 ± 3 a	
		1:50	146 ± 1 b-d	186 ± 2 ab	104 ± 1 a-c	
		1:100	138 ± 1 de	161 ± 0 c	79.1 ± 2.6 d-g	
		1:250	125 ± 1 f-h	154 ± 2 c	74.5 ± 1.4 e-g	
		1:500	116 ± 2 hi	140 ± 2 de	82 ± 1.9 c-g	
	90	1:25	158 ± 1 a	188 ± 1 ab	108 ± 2 ab	

		1:50	161 ± 1 a	193 ± 1 a	105 ± 2 a-c	
		1:100	153 ± 1 a-c	185 ± 2 ab	86.7 ± 6 b-g	
		1:250	155 ± 2 ab	182 ± 2 ab	69.3 ± 5 f-h	
		1:500	159 ± 1 a	180 ± 2 b	79.1 ± 1 d-g	
Luteolin-7-*O*-glucoside	30	1:25	14 ± 1.5 hi	15.6 ± 0.8 j-l	4.98 ± 3.5 k	***
		1:50	13.2 ± 1.9 hi	17.5 ± 1.1 i-l	8.23 ± 0.43 jk	
		1:100	26.6 ± 3.3 e-h	37.9 ± 3.6 b-d	17.7 ± 0.7 f-j	
		1:250	24.7 ± 3.4 e-h	22.4 ± 2.9 g-k	18.3 ± 2.3 f-j	
		1:500	16.2 ± 1.3 g-i	13.9 ± 0.9 kl	23.2 ± 0.2 e-g	
	45	1:25	15.3 ± 0.4 hi	15.2 ± 1.1 j-l	21.8 ± 2.4 e-h	
		1:50	14.6 ± 2.7 hi	18 ± 1 h-l	28.6 ± 1.7 d-f	
		1:100	26.5 ± 4.2 e-h	33.4 ± 2.1 d-f	36.5 ± 3.1 b-d	
		1:250	12.8 ± 0.6 hi	14.5 ± 0.6 kl	20.2 ± 2.9 e-i	
		1:500	7.73 ± 0.78 i	16.1 ± 0.9 j-l	9.1 ± 3.73 i-k	
	60	1:25	22.9 ± 0.3 f-h	30.8 ± 1.4 d-g	41.9 ± 1.8 bc	
		1:50	32.1 ± 0.8 ef	47.1 ± 2.9 ab	29.1 ± 0.9 d-f	
		1:100	22.8 ± 2.3 f-i	27.9 ± 0.6 d-h	18.4 ± 0.9 f-j	
		1:250	19.2 ± 0.2 f-i	14.2 ± 0.3 kl	8.42 ± 1.34 i-k	
		1:500	22.8 ± 1 f-h	10.3 ± 0.6 l	9.73 ± 0.19 i-k	
	75	1:25	38.6 ± 1.8 de	27 ± 1.6 e-i	30.9 ± 0.7 c-e	
		1:50	54.3 ± 0.9 c	29.1 ± 4.3 d-g	42.2 ± 1.1 bc	
		1:100	31.1 ± 1 e-g	35.8 ± 1.3 c-e	29.3 ± 0.1 d-f	
		1:250	20.6 ± 0.7 f-i	24.8 ± 1.9 f-j	15.6 ± 1.1 g-k	
		1:500	12.7 ± 2.2 hi	10.7 ± 0.7 l	9.93 ± 0.62 h-k	
	90	1:25	78.9 ± 7.5 a	45.9 ± 1.7 a-c	37.5 ± 6.3 b-d	

		1:50	69.7 ± 1.2 ab	29.2 ± 0.5 d-g	57.2 ± 2.2 a	
		1:100	74 ± 1.8 a	55.2 ± 1.8 a	48.2 ± 1.5 ab	
		1:250	55.6 ± 6.1 bc	18.5 ± 1.2 h-l	29 ± 1.4 d-f	
		1:500	51.6 ± 4.1 cd	44.3 ± 3 bc	29.6 ± 0.3 d-f	
Apigenin-7-glucoside	30	1:25	25.7 ± 1.4 l	30.2 ± 1.9 l	3.29 ± 3.29 i	***
		1:50	27 ± 2.4 kl	30.6 ± 1.6 l	28.7 ± 4 gh	
		1:100	46.4 ± 2.9 f-j	51.7 ± 2.1 d-i	37.2 ± 2.6 f-h	
		1:250	47.4 ± 3.4 e-j	41.4 ± 1.6 jk	27.9 ± 2.4 h	
		1:500	41.3 ± 1.7 h-j	41.1 ± 0.8 jk	49.8 ± 1.2 a-f	
		1:25	38.5 ± 0.7 ij	29.2 ± 0.8 l	38.3 ± 1.9 e-h	
		1:50	41.3 ± 3.1 h-j	35.1 ± 1.8 kl	48 ± 1.6 a-f	
	45	1:100	54.3 ± 3 d-g	55.9 ± 1.5 c-f	59.8 ± 2 a	
		1:250	52.8 ± 0.8 e-g	41.4 ± 0.8 jk	53.9 ± 3.3 a-d	
		1:500	45.5 ± 2.3 g-j	46.7 ± 2.2 g-j	49.5 ± 6.3 a-f	
	60	1:25	37.7 ± 0.4 jk	46.1 ± 0.3 h-j	56.4 ± 0.9 a-c	
		1:50	49.5 ± 0.9 e-h	61.5 ± 1.3 bc	51.2 ± 0.9 a-e	
		1:100	46 ± 1 f-j	59.9 ± 0.8 b-d	48.5 ± 1.4 a-f	
		1:250	44.6 ± 1.1 g-j	52.2 ± 0.4 d-i	39.1 ± 2.7 e-h	
		1:500	50.5 ± 1.2 e-h	47.7 ± 0.7 f-j	41.9 ± 0.2 d-g	
	75	1:25	46.3 ± 1.1 f-j	48.2 ± 1.1 e-j	45.5 ± 0.4 b-f	
		1:50	69.5 ± 1.5 ab	50.2 ± 4.6 e-i	58.7 ± 0.8 ab	
		1:100	58.1 ± 0.2 c-e	59.3 ± 0.6 b-d	55.3 ± 0.4 a-c	
		1:250	56.3 ± 0.6 d-f	55.1 ± 1.9 c-g	44.5 ± 0.4 c-f	
		1:500	48.9 ± 3.2 e-i	48.5 ± 1 e-j	40.8 ± 1 d-h	
	90	1:25	69.8 ± 2.2 ab	54 ± 0.9 c-h	44.1 ± 5.4 c-f	

		1:50	77.9 ± 0.2 a	45.1 ± 0.4 ij	59.1 ± 1 a	
		1:100	73.8 ± 0.3 ab	70.3 ± 1.2 a	58.9 ± 1.3 a	
		1:250	67.5 ± 3.5 a-c	56.8 ± 2 b-e	49.1 ± 1 a-f	
		1:500	64.7 ± 2.1 b-d	64.8 ± 1.1 ab	53.5 ± 1.1 a-d	
Oleuropein	30	1:25	517 ± 4 n	450 ± 26 l	167 ± 11 j	***
		1:50	658 ± 49 lm	655 ± 6 k	197 ± 6 j	
		1:100	848 ± 13 jk	807 ± 6 ij	507 ± 59 f-i	
		1:250	857 ± 51 jk	839 ± 39 i	682 ± 47 c-h	
		1:500	980 ± 49 ij	711 ± 30 jk	1218 ± 122 a	
	45	1:25	599 ± 4 mn	471 ± 6 l	726 ± 1 b-g	
		1:50	761 ± 9 kl	641 ± 9 k	795 ± 5 b-f	
		1:100	788 ± 23 kl	719 ± 15 jk	809 ± 13 b-e	
		1:250	951 ± 16 ij	809 ± 8 ij	940 ± 26 a-c	
		1:500	1174 ± 21 gh	1032 ± 54 h	774 ± 40 b-f	
	60	1:25	727 ± 15 k-m	1065 ± 9 gh	618 ± 31 d-i	
		1:50	930 ± 4 ij	1267 ± 16 f	539 ± 16 e-i	
		1:100	1050 ± 28 hi	1401 ± 3 e	446 ± 4 g-j	
		1:250	1252 ± 5 g	1648 ± 21 c	382 ± 52 ij	
		1:500	1499 ± 47 ef	1960 ± 20 a	752 ± 15 b-f	
	75	1:25	1186 ± 6 g	869 ± 3 i	822 ± 24 b-e	
		1:50	1456 ± 12 f	1145 ± 17 g	954 ± 17 a-c	
		1:100	1628 ± 13 de	1283 ± 2 f	920 ± 35 bc	
		1:250	1986 ± 34 c	1541 ± 21 cd	870 ± 40 b-d	
		1:500	2213 ± 12 b	1895 ± 28 ab	877 ± 86 b-d	

	90	1:25	1269 ± 7 g	882 ± 7 i	983 ± 27 ab	
		1:50	1580 ± 5 ef	1089 ± 2 gh	1002 ± 22 ab	
		1:100	1758 ± 6 d	1286 ± 7 f	915 ± 79 bc	
		1:250	2123 ± 18 b	1534 ± 10 d	749 ± 125 b-f	
		1:500	2397 ± 17 a	1824 ± 23 b	412 ± 90 h-j	
Sum of polyphenols	30	1:25	852 ± 4 o	785 ± 25 m	212 ± 17 l	***
		1:50	1081 ± 42 n	971 ± 6 l	496 ± 26 kl	
		1:100	1353 ± 4 j-l	1279 ± 15 j	862 ± 61 h-j	
		1:250	1327 ± 59 j-l	1288 ± 39 j	953 ± 50 f-j	
		1:500	1450 ± 58 i-k	1110 ± 27 k	1582 ± 122 a	
	45	1:25	1019 ± 6 n	824 ± 3 m	1076 ± 7 d-i	
		1:50	1230 ± 9 lm	1045 ± 12 kl	1208 ± 7 b-g	
		1:100	1314 ± 37 kl	1227 ± 14 j	1268 ± 20 b-e	
		1:250	1461 ± 18 ij	1243 ± 4 j	1370 ± 34 a-d	
		1:500	1662 ± 29 gh	1539 ± 57 g-i	1176 ± 45 b-g	
	60	1:25	1156 ± 16 mn	1568 ± 8 gh	1053 ± 38 e-i	
		1:50	1447 ± 10 i-k	1860 ± 14 e	901 ± 17 g-j	
		1:100	1587 ± 32 hi	1966 ± 1 de	765 ± 10 i-k	
		1:250	1766 ± 6 g	2203 ± 39 b	700 ± 58 jk	
		1:500	2037 ± 55 f	2476 ± 22 a	1108 ± 17 c-h	
	75	1:25	1707 ± 3 gh	1437 ± 8 i	1227 ± 30 b-f	
		1:50	2096 ± 11 ef	1711 ± 20 f	1396 ± 17 a-c	
		1:100	2221 ± 13 de	1850 ± 3 e	1311 ± 31 a-e	
		1:250	2544 ± 33 c	2076 ± 22 cd	1207 ± 35 b-g	

	1:500	2727 ± 11 b	2383 ± 29 a	1227 ± 93 b-f
90	1:25	1959 ± 8 f	1475 ± 6 hi	1398 ± 56 a-c
	1:50	2270 ± 2 de	1605 ± 2 fg	1460 ± 25 ab
	1:100	2435 ± 10 c	1900 ± 10 e	1339 ± 88 a-e
	1:250	2748 ± 9 b	2099 ± 9 bc	1104 ± 142 c-h
	1:500	3037 ± 22 a	2405 ± 22 a	781 ± 84 i-k

MAC – maceration; UAE – ultrasound-assisted extraction; MAE – microwave-assisted extraction; TE – Trolox equivalents; *– p-value ≤ 0.05; ** – p-value ≤ 0.01; *** – p-value ≤ 0.001. Small letters represent different homogenous groups using Tukey's post-hoc test.
